# Supplementary material for: The BCC7 Protein Contributes to the Toxoplasma Basal Pole by Interfacing between the MyoC Motor and the IMC Membrane Network
Source: Int J Mol Sci. 2022 May 26;23(11):5995. doi: 10.3390/ijms23115995 (PMC9181098; doi:10.3390/ijms23115995)
Supplement: Supplementary file 1 [file ijms-23-05995-s001.zip › Vigetti-Laboure_Original-Blots.pdf]

Article

The BCC7 protein contributes to the *Toxoplasma* basal pole by interfacing between the MyoC motor and the IMC membrane network

Luis Vigetti[1,†], Tatiana Labouré[1,†], Chloé Roumégous[2], Dominique Canella[3], Bastien Touquet[1], Claudine Mayer[4,5], Yohann Couté[6], Karine Frénal[2], Isabelle Tardieux[1,*] and Patricia Renesto[1,*]

Original Blots images

Figure 1 b

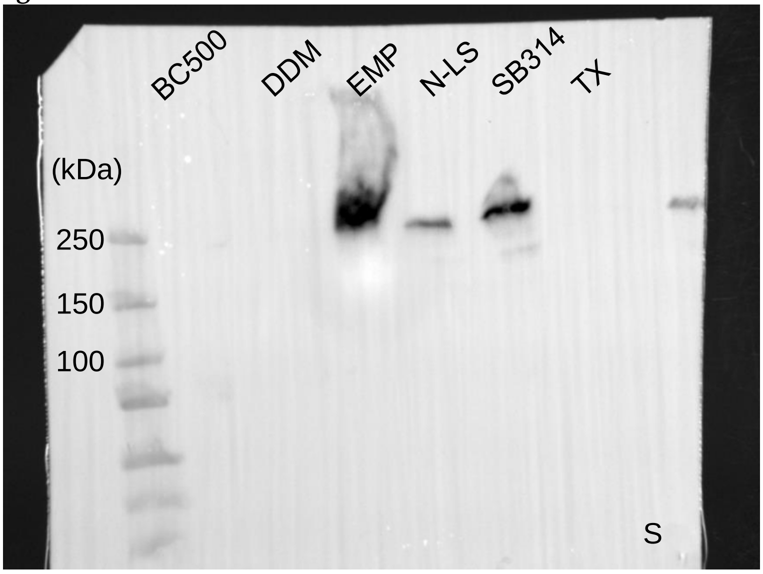

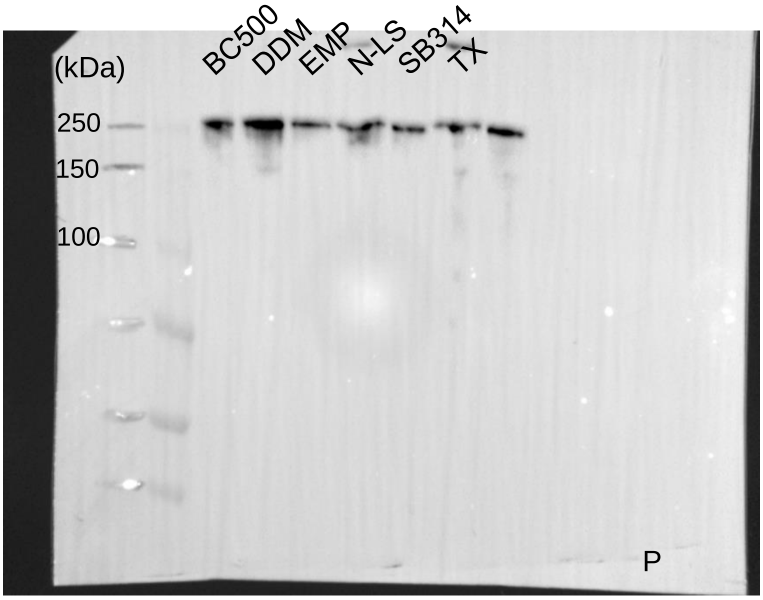

Western Blot corresponding to Figure 1b, with the soluble (upper) and insoluble (lower) fractions from each lysate.

BC500: control lysis buffer containing NP40 (0.05% v/v)

DDM: BC500 supplemented with n-Dodecyl-β-D-maltopyranoside (0.1 % w/v)

EMP: Empigen BB (1% v/v)

N-LS: N-lauryl sarcoside (0.1% w/v)

SB314: sulfobetaine 3-14 (n-Tetradecyl-N,N-dimethyl-3-ammonio-1-propanesulfonate) (1% w/v)

TX: Triton X-100 (0.1% v/v).

Figure S3 a

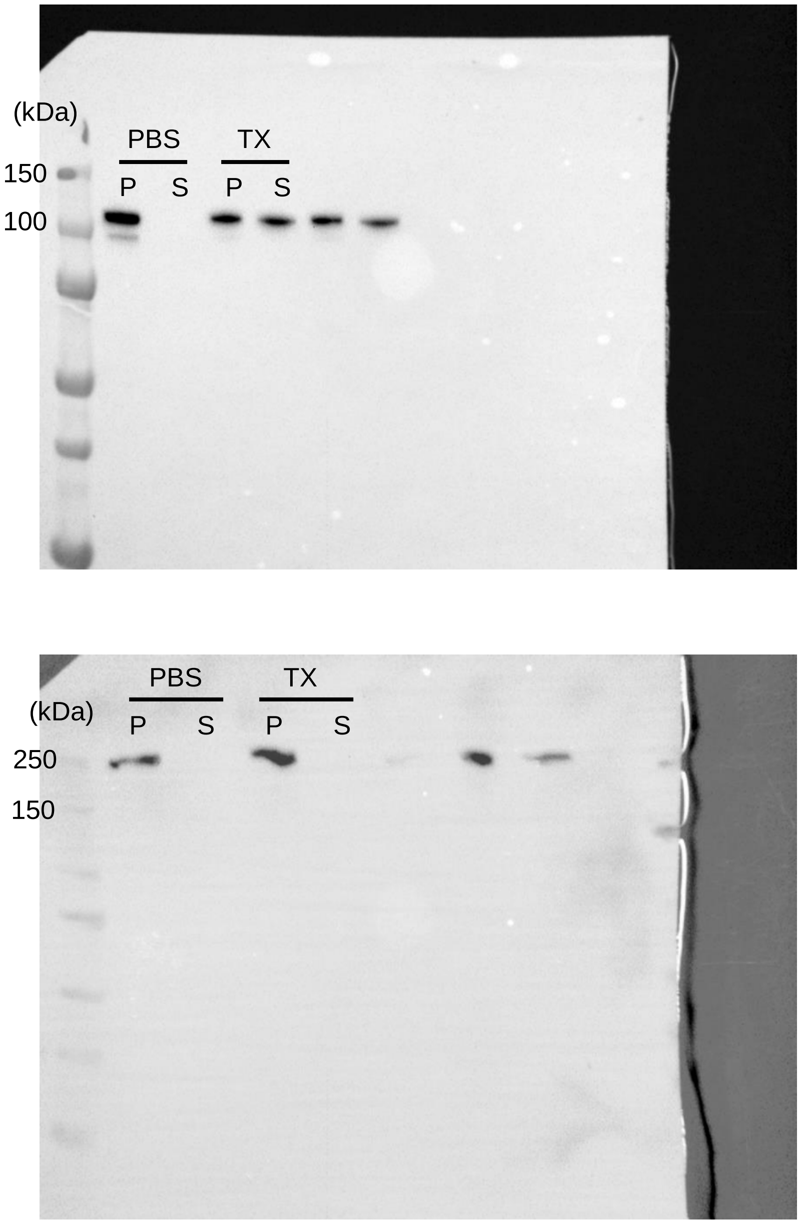

Western-blot analysis of insoluble (P) and soluble (S) fractions recovered following cell disruption in PBS or Triton X-100 1% v/v (TX) of RH◎Ku80 strains expressing endogenously HA-tagged MyoA (upper) or BCC7 (lower panel). These images are representative of 2 distinct experiments.

Figure S3 b

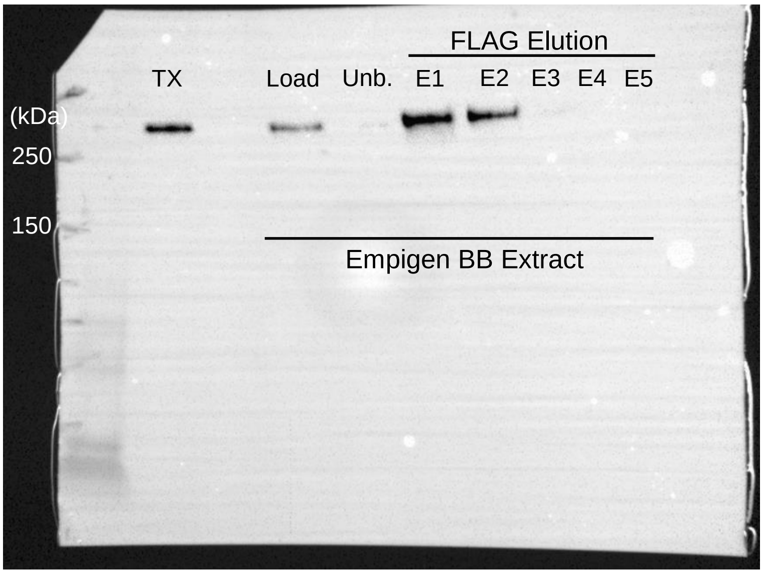

The TX-insoluble proteins were further extracted with Empigen BB. The Empigen BB supernatant that contained BCC7-HA-FLAG (Load) was incubated with anti-FLAG antibodies immobilized on agarose resin. The protein was competitively eluted with FLAG peptides (E1 to E5). Aliquots of each fraction were resolved on SDS-PAGE, and probed with anti-HA antibodies. The molecular weights are indicated in kilodaltons (kDa) on the left. This image is representative of 7 distinct experiments.
