# Supplementary figures and images for: The BCC7 Protein Contributes to the Toxoplasma Basal Pole by Interfacing between the MyoC Motor and the IMC Membrane Network
Source: Int J Mol Sci. 2022 May 26;23(11):5995. doi: 10.3390/ijms23115995 (PMC9181098; doi:10.3390/ijms23115995)

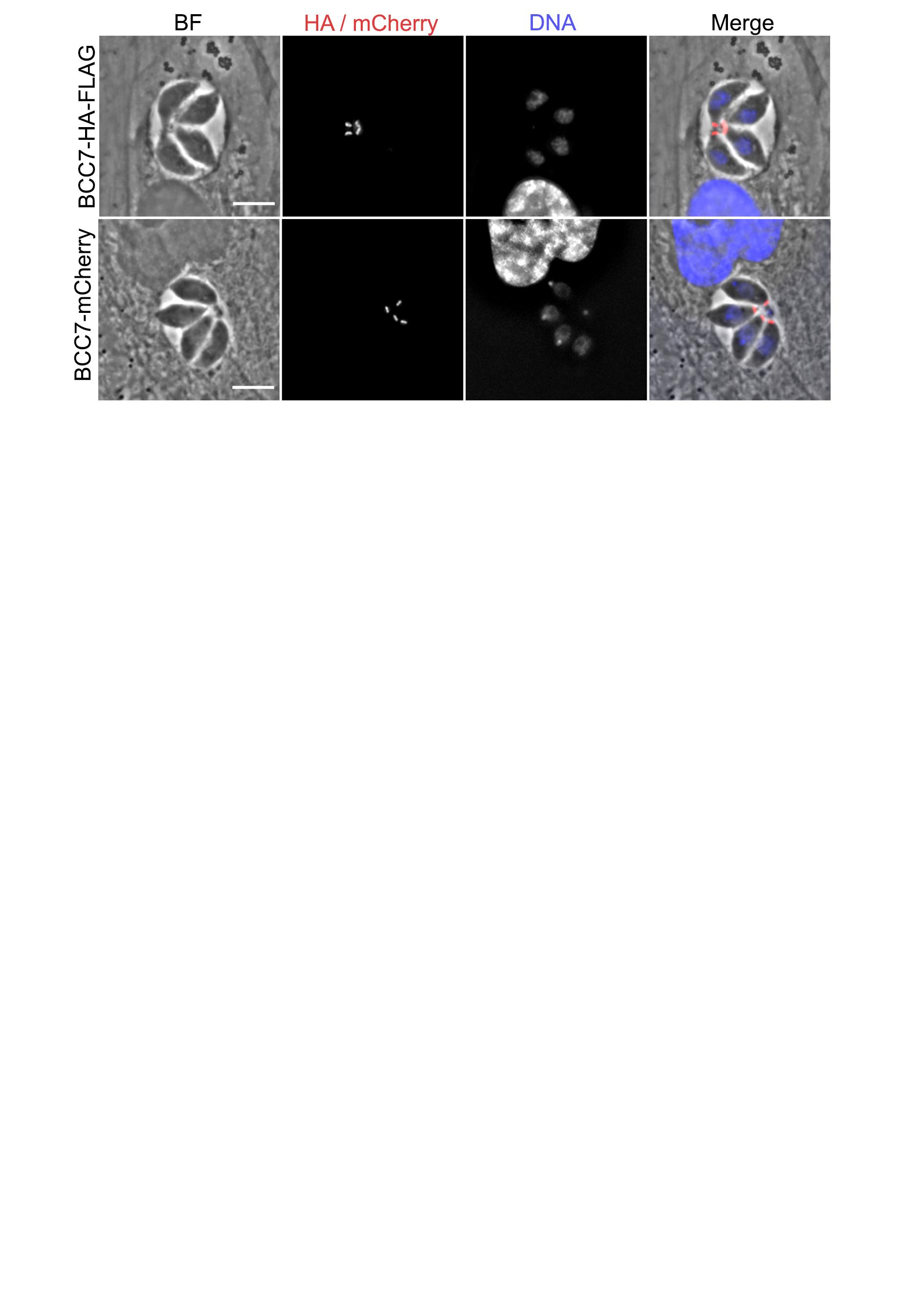

Supplement: Supplementary file 1 [file ijms-23-05995-s001.zip › Figure S1.tif]

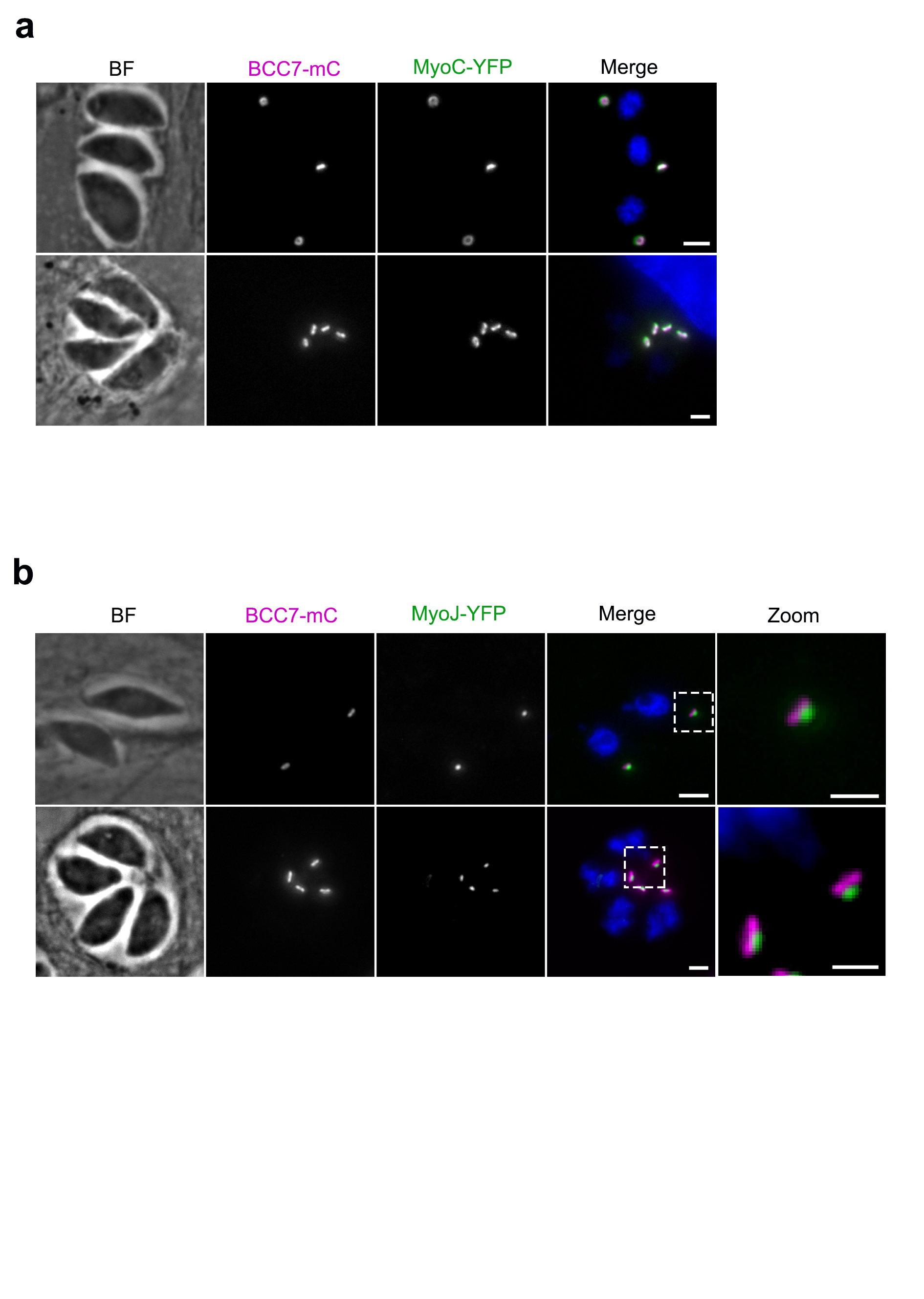

Supplement: Supplementary file 1 [file ijms-23-05995-s001.zip › Figure S2.tif]

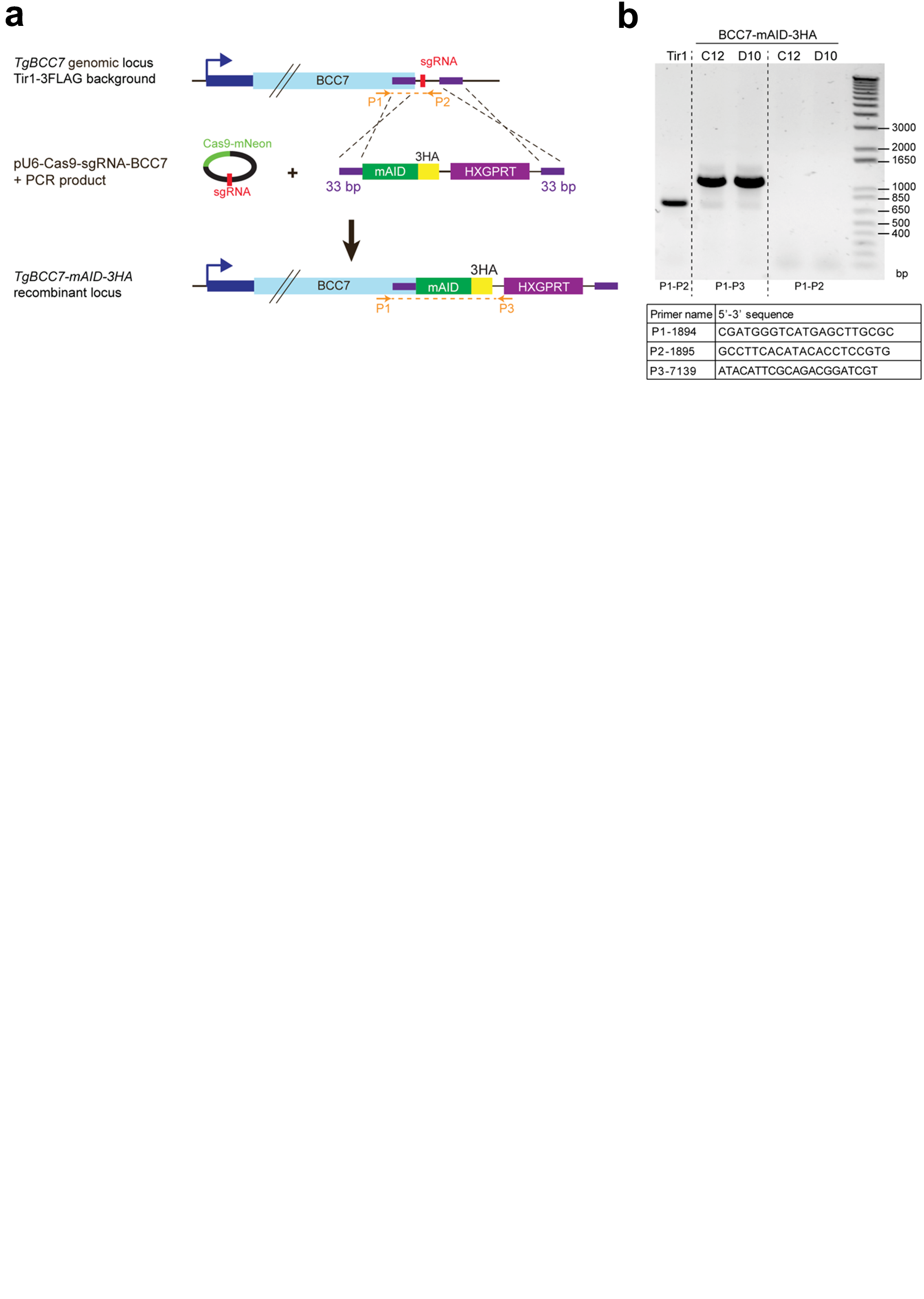

Supplement: Supplementary file 1 [file ijms-23-05995-s001.zip › Figure S3.tif]

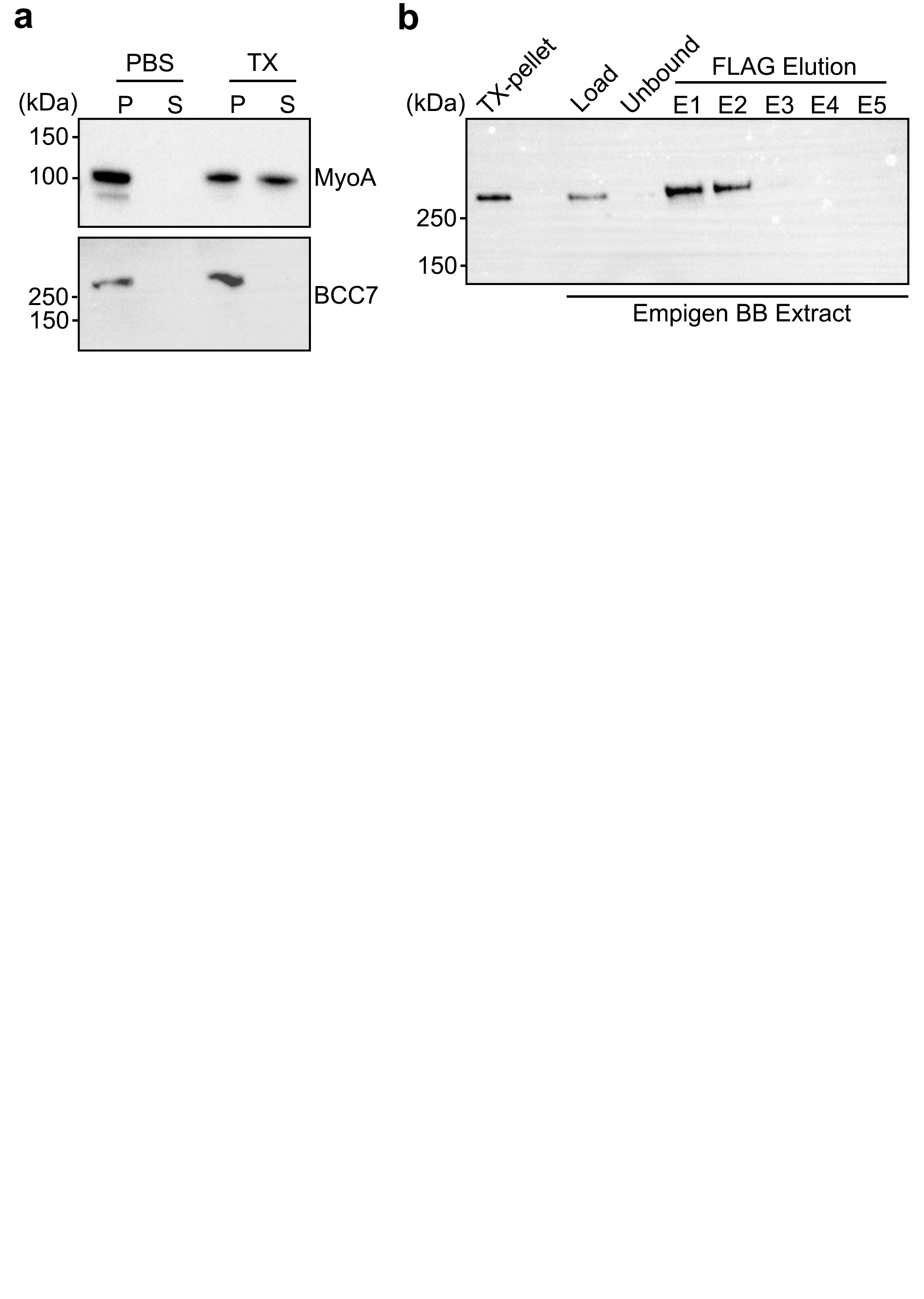

Supplement: Supplementary file 1 [file ijms-23-05995-s001.zip › Figure S4.tif]

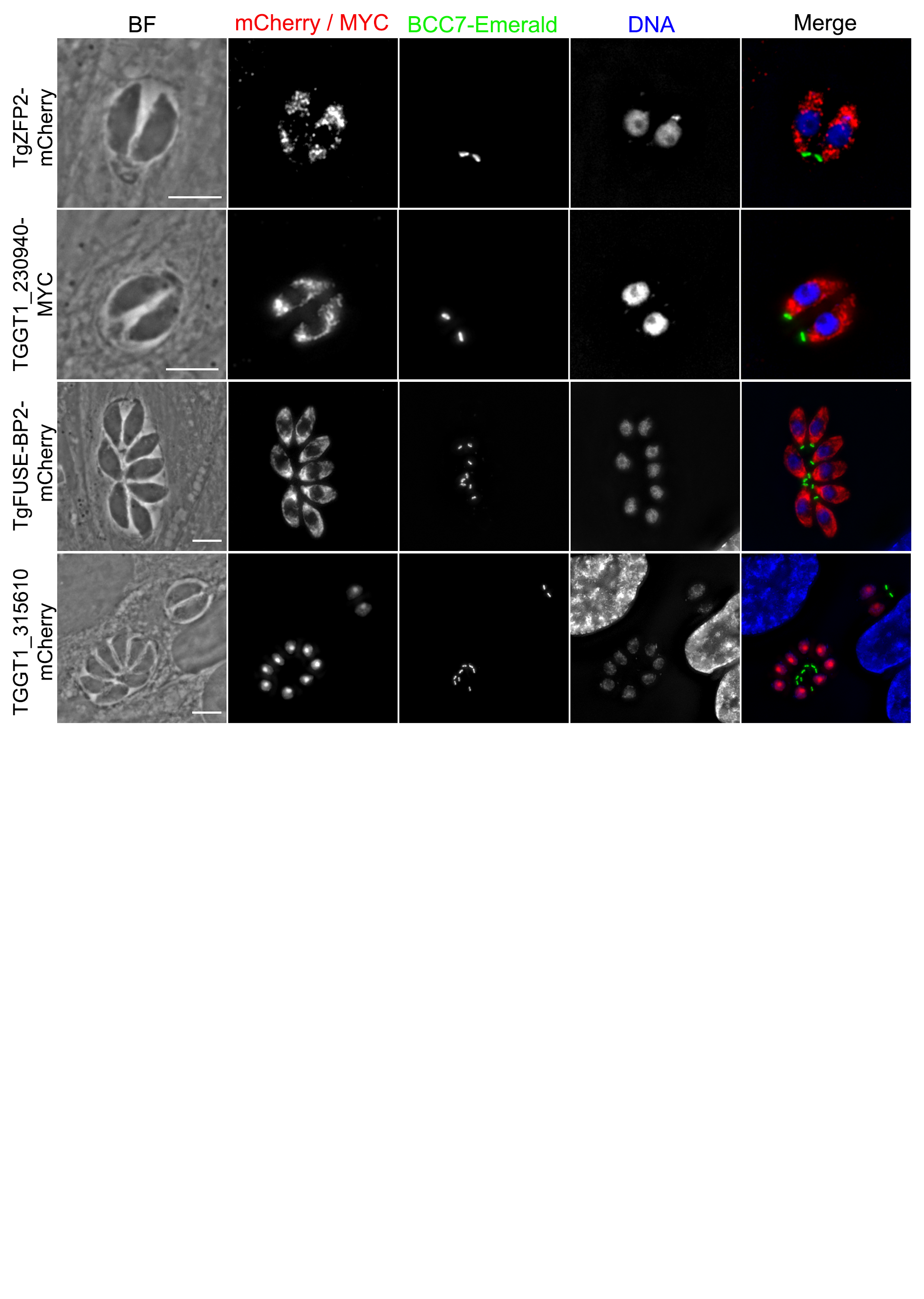

Supplement: Supplementary file 1 [file ijms-23-05995-s001.zip › Figure S5.tif]
